# Long-term treatment with low-dose medicine in chronic childhood eczema: a double-blind two-stage randomized control trial

**DOI:** 10.1186/s13052-017-0393-5

**Published:** 2017-09-06

**Authors:** R. Carello, L. Ricottini, V. Miranda, P. Panei, L. Rocchi, R. Arcieri, E. Galli

**Affiliations:** 1 Department of Pediatric Allergy, San Pietro Hospital Fatebenefratelli, Via Cassia, 600 Rome, Italy; 2 Clinical Research Unit, Guna S.p.a., Milan, Italy; 3 Department of Pharmacology and Therapeutic Research, Italian National Institute of Health, Rome, Italy

**Keywords:** Eczema, Immunomodulation, Low-dose medicine, SCORAD index

## Abstract

**Background:**

The efficacy of low-dose medicine (LDM) in childhood mild/moderate eczema is not known. We conducted a double-blind, two-stage, randomized, placebo-controlled clinical trial, lasting 23 months, to address this issue.

**Method:**

Eighty children with chronic mild/moderate eczema were randomly allocated to Group A (placebo) or Group B (treatment group; Galium-Heel®, a low-dose multicomponent medicine based upon natural substances; Guna-Interleukin 12 and Guna-Interferon-γ administered twice a day for six non-consecutive months for each stage). LDM is characterized by the use of biological molecules, such as cytokines, neuropeptides, growth factors, hormones at very low concentrations, which correspond to physiological levels within the human body. The dosage of the cytokines used in this trial (IFN-γ and IL-12) is 10 fg/ml. The SCORAD index was evaluated by the same operator: subjects with a SCORAD index below 20 were considered to have mild eczema (61/80; mean: 10.79), whereas a SCORAD index between 20–50 indicated moderate eczema (19/80; mean: 26.84). The data of 66/80 children were analyzed in stage 1 and those of 62/66 children in stage 2. The primary outcome measure was reduction of eczema severity assessed by the SCORAD index. Secondary outcomes were disease-free interval, and treatment safety and tolerability.

**Results:**

The decrease in disease severity was greater in Group B than in Group A already in stage 1 (a decrease 63.9% versus 53.2%), but the difference was not significant (*p* = 0.16). Moreover, subjective symptoms (itching and sleep disturbances) initially decreased and then worsened in Group A, whereas itching decreased linearly and sleep disturbances decreased significantly (*p*=0.049) in Group B.

**Conclusions:**

Preliminary evidence suggests potential benefit, but further work is needed to validate this approach.

**Trial registration:**

The trial was registered with EudraCT number 2010–018640-13 through the database of the National Clinical Trials Monitoring Centre Database (Osservatorio delle Sperimentazioni Cliniche, OsSC) of the Italian Medicines Agency.

## Background

Eczema is the most frequent chronic inflammatory condition in childhood; indeed it affects up to 20% of children and starts in the first years of life [1–4]. Genetic, epigenetic and environmental factors, and disturbed skin barrier function as well as innate and adaptive immune defects, are associated with eczema onset, although the pathogenic mechanisms are not fully understood [2, 3, 5]. It is a frustrating condition for both patients and caregivers because intense pruritus and sleep disturbances can be intractable, and can negatively impact on school, social and family life thereby resulting in psychological difficulties for all the family, and a high socioeconomic burden [6, 7]. Conventional treatment of moderate-severe eczema may not be sufficient to control the activity of skin lesions, and a small subset of children require more aggressive systemic therapy which is not without side effects [8–10].

Low-dose medicine (LDM) is a new therapeutic approach theorized and developed starting from the most recent knowledge in molecular biology, psycho-neuro-endocrine-immunology, and research results in the field of low dose pharmacology. The most intriguing aspect of LDM is the efficacy of oral administration of low dose “signaling molecules”. The use of physiological low doses (predominantly nanograms-femtograms) *per os* in LDM is made possible by the application of SKA (*Sequential Kinetic Activation*) technology [11, 12]. SKA is a sophisticated drug delivery system, developed according to a pharmaceutical techniques based upon a mechanical process.

The action mechanism of SKA low dose cytokines consists in sensitization and activation of some units of cellular (or plasmatic) receptors in virtue of their low concentration, practically in their physiological working range between 10^-12^ M and 10^-15^ M [13]. LDM exerts pharmacomodulatory activity that can restore the homeostatic balance between the various lymphocyte populations, particularly in allergic and inflammatory diseases. In Gariboldi et al., low-dose concentrations of IL-12 and IFN-γ have been used in the in vivo treatment of experimental allergic bronchial asthma (in an animal model) with very encouraging results [14]. Cardani et al. demonstrated that oral administration of low-dose IL-10 and Ab anti-IL-1 is able to control intestinal inflammation in an in vivo animal model of IBD (Inflammatory Bowel Disease) [15]. Roberti ML et al. demonstrated the immunomodulatory activity of IL-4, IL-10, and IL-11 in psoriasis vulgaris [16].

The purpose of our double-blind, two-stage randomized clinical trial was to assess the clinical efficacy and safety of long-term LDM treatment in children affected by mild/moderate chronic eczema.

## Methods

### Study design

**Fig. 1:**
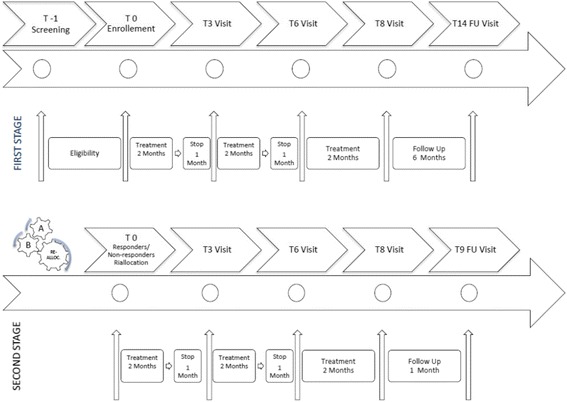
Visits and treatment during the first and second stages of the study

**Fig. 2:**
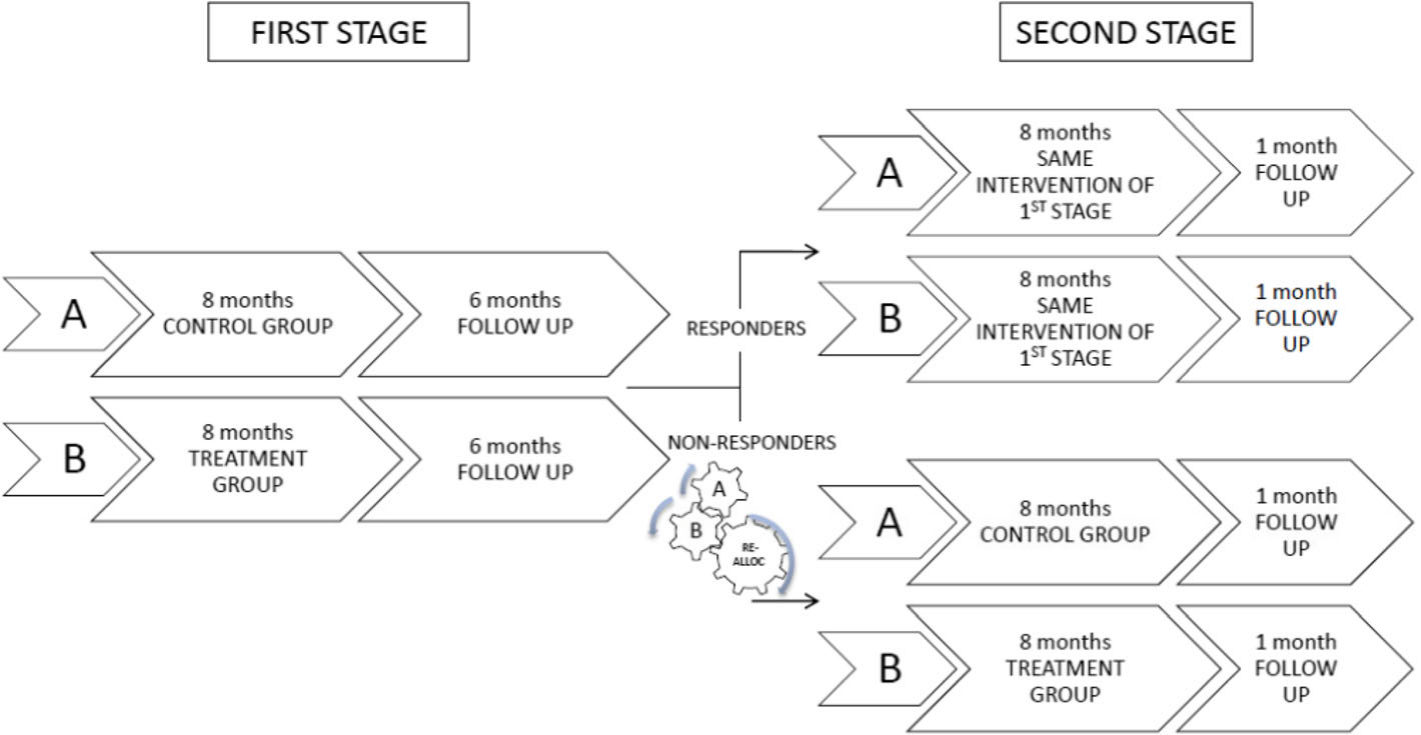
Study design. After stage 1, responders (identified by clinical evaluation, received the same intervention during stage 2 (AA; BB). Non-responders were randomly allocated to treatment or placebo: A and B that become AA; BB; AB; BA. AA, children who took placebo in both stage 1 and 2 of the study. BB, children who took LDM in both stage 1 and 2 of the study. AB, children who assumed placebo in stage 1 and LDM in stage 2. BA, children who treated with LDM in stage 1 and placebo in stage 2

The aim of this double-blind, two-stage, randomized, placebo-controlled clinical trial was to evaluate the efficacy and safety of long-term treatment (23 months) with LDM *vs* placebo in children with chronic mild/moderate eczema. Patients were randomized into either Group A (placebo group) or Group B (treatment group). In the first stage, all randomized children assumed LDM or placebo for 6 non consecutive months over a period of 8 months, followed by a 6 month follow-up (Fig. 1). In the second stage, non-responders to LDM were re-randomized to receive either LDM or placebo with the same modality as in the first stage, followed by a one-month follow-up (Fig. 2), whereas responders continued the same treatment (LDM or placebo) as in the first stage of the study.

Children in an acute phase of the disease (see below) were screened by the Clinical Screening Operative Unit, which is constituted by pediatricians operating in the Lazio region, and enrolled at the Department of Pediatric Allergy, San Pietro Hospital Fatebenefratelli (Rome, Italy). Inclusion criteria were children affected by chronic mild/moderate eczema (SCORAD index: <6 − <40), with at least 4 relapses per year, and with onset of skin lesions at least 6 months before the study (all children were in an acute phase of the disease upon enrolment); children with IgE-mediated eczema (i.e., children who tested positive to specific in vivo and/or in vitro tests, and children with non-IgE-mediated eczema (negative to specific in vivo and/or in vitro tests); and written informed consent of parents and guardians to the study. Exclusion criteria were: systemic treatment with corticosteroids and antihistamines, with topical calcineurin inhibitors (tacrolimus and/or pimecrolimus) or with specific immunotherapy in the three months before the study; and severe eczema-associated systemic disorders.

### Patients

**Fig. 3:**
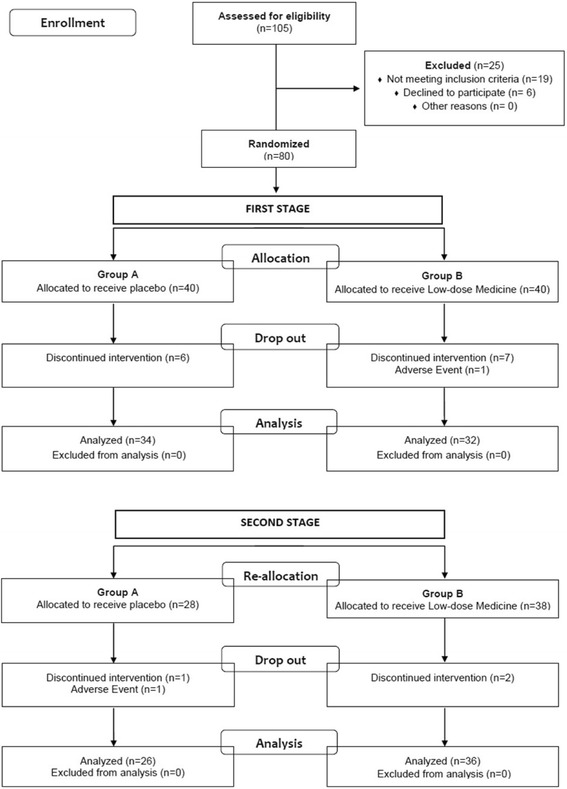
Clinical trial flow diagram (Consort 2010) of the first and second stage of the study

Children were enrolled at the Department of Pediatric Allergy, San Pietro Hospital Fatebenefratelli, Rome between February 2010 and July 2013. A total of 105 children were screened and 80 were enrolled and randomized as shown in the clinical trial flow diagram (Fig. 3 and Table 1). In detail, 43 boys and 37 girls (mean age: 73.77 months; range: 18 months − 16 years) with mild/moderate chronic eczema, diagnosed according to the revised Hanifin and Rajka criteria [17], were enrolled in the study. Eczema was considered chronic if it lasted at least 6 months (relapses ≥4/year). Subjects in remission during screening were excluded.

**Table 1:** Distribution of epidemiological and clinical characteristics at randomization

Variable	Group A	Group B	*p* value
SCORAD index (mean value) ± SD	13.93 ± 8.28	14.71 ± 7.64	0.66
% Boys	55.0	52.5	> 0.99
Mean age (months) ± SD	69.0 ± 43.6	78.6 ± 50.9	0.37
Median age (months)	64.5	60.5	
Age at diagnosis (months)	51.2	68.2	0.48
IgE kU/l (mean value) ± SD	373.5 ± 929.4	409.6 ± 561.4	0.83
% positive prick test	42.5	47.5	0.58

Disease severity was assessed with the Scoring Atopic Dermatitis (SCORAD) index that rates the extent and intensity of eczema, and subjective symptoms, namely pruritus and sleeplessness [18]. The SCORAD index was evaluated by the same operator (R.C.) throughout the study. Subjects with a SCORAD index below 20 were considered to have mild eczema (61/80; mean: 10.79), whereas a SCORAD index between 20 and 50 indicated moderate eczema (19/80; mean: 26.84). None of the enrolled children suffered from other chronic diseases or was taking oral steroids, antihistamines or topical calcineurin inhibitors, specific immunotherapy or immunosuppressive therapy at the time of investigation.

The data of each child, including adverse events, were recorded on an electronic case report form at each visit. At time zero (T0), each patient was given a clinical diary on which to record to symptomatology and the use, if any, of symptomatic treatment during the acute phase.

### Treatment

SKA-cytokines were prepared by GUNA Laboratories (GUNA S.p.a., Milan, Italy) using the standardized method [14]. IFN-γ and IL-12 were activated by sequential serial dilution (1:100) in 30% hydro-alcoholic solution and kinetically energized by a shaking procedure (vertical shaking; 10 cm motion range; shaking speed corresponding to 100 oscillations in 10 s). Subjects who satisfied the eligibility criteria were randomly allocated to Group A or Group B, and identified by their initials and a serial number. Group B (treatment group) was treated with Galium-Heel® (Biologische Heilmittel Heel GmbH, Baden Baden, Germany), Guna-Interleukin 12 and Guna-Interferon γ at the concentration of 10 fg/ml SKA [11, 14, 19] in hydro-alcoholic solution 30%. Each preparation was orally administered (8 drops in children aged ≤5 years and 15 drops in children above the age of 5) twice daily for 6 non-consecutive months in each stage. This concentration is far from being toxic, therefore the concept of pharmacological use in terms of “pro-kg amount” would be nonsense. Group A received placebo (hydro-alcoholic solution 30% without active ingredients) with the same mode of administration.

During the study, all children received conventional therapy (topical steroids, topical or oral antibiotics and antihistamines) when necessary to alleviate symptoms.

### Outcomes

The primary outcome measure was a decrease in severity based on the SCORAD index. Secondary outcomes were disease-free interval, treatment safety and tolerability, skin prick test for inhalant and food allergens, skin prick-by-prick, total and specific IgE assessment and serum cytokine level of IL-4, IL-10, IL-12, IL-13, IFN-γ.

### Sample size

The required sample size was estimated with respect to the variation in the SCORAD index, based on the paired Student’s t test two-tailed comparison of the mean value between Groups A and B at baseline, and 3, 6, 8 and 14 months thereafter. We recorded differences in the SCORAD index in terms of increase and decrease, and therefore we considered both improvements (a decrease in the SCORAD index) and worsening (an increase in the SCORAD index). A difference of 30% between baseline to 14 months was considered clinically relevant. Finally, assuming a power 1-β = 0.80 and a probability of a type I error α = 0.05, the required sample size was 128.

### Randomization

Children who satisfied the eligibility criteria were randomized using software with a 128 random list for the two groups (A and B) in a 1:1 ratio. Block randomization was performed with a block size of 16. The subjects were assigned to Group A or Group B, according to the sequential order of enrolment. A second 128 random list was generated for the second stage of the study (Fig. 2). The random allocation sequence was generated by biostatisticians (Department of Pharmacology and Therapeutic Research, Italian National Institute of Health, Rome, Italy).

### Sample collection and methods

The timing of sampling is shown in Fig. 1. IL-4, IL-10, IL-12, IL-13 and IFN-γ were quantified by a double-antibody sandwich ELISA method according to the manufacturer’s instructions (Thermo Scientifics, Waltham, MA, USA). Skin prick tests were performed and interpreted as previously described [20] with commercial inhalant and food allergens (Stallergenes, Milan, Italy). Prick-by prick-tests were performed directly with the offending food. Total serum IgE and allergen-specific IgE were quantified in the sera of subjects with a fluorescence enzyme immunoassay ImmunoCAP (Phadia/Thermo Fisher Scientific Inc., Uppsala, Sweden) in accordance with the product manual with a lower detection limit of 0.35 kU/l and a maximum limit of 100 kU/l.

### Statistical analysis

The data were analyzed with SPSS 22 and OpenEpi 3.02 software packages. Categorical variables are presented as absolute and percent frequencies, and quantitative variables are showed as means ± standard deviations. The data were analyzed separately from T0 to T3, from T3 to T6, from T6 to T8, and from T8 to T14 to assess the efficacy of the treatment in reducing the SCORAD index (“absolute effectiveness”). We also compared the two groups at different times to evaluate the relative effectiveness of the two treatments. We calculated the probability (odds ratio; OR) of the disease-free interval between the study groups, using person/time to normalize the difference in the number of subjects in each group, and the difference in time of the presence of each subject in the study. We calculated the OR of being a non-responder to therapy by comparing Group A and Group B. For any reference period, only subjects with data at baseline and at least one observation during the study were included in the analysis. We performed an intention-to-treat analysis and per-protocol analysis. An interim analysis was carried out at the end of the first phase of the study (data not shown).

## Results

As shown in the flowchart (Fig. 3), 66 of the 80 enrolled children were included in the statistical analysis of the first stage of the study: 40 in Group A and 40 in Group B. There were 14 drop-outs (17.5%) in this stage. Thirteen discontinued medication (6 in Group A and 7 in Group B), and one child in Group B experienced an adverse event (Fig. 3). In the second stage of the study, 4 (7.1%) children discontinued the intervention (2 in Group A and 2 in Group B). One child in Group A went abroad and the other experienced an adverse event. The two children in Group B discontinued for non compliance as indicated by their parents. There were no significant intergroup differences in terms of gender, age and clinical findings (Table 1).

### SCORAD analysis

In the first stage of the study, the SCORAD index decreased between T0 and T14 in both groups. In Group A (placebo) it decreased by 41.5% between T0 and T8 (*p* = 0.001), and by 53.2% between T0 and T14 (*p* < 0.001). In Group B (treatment), it decreased by 53.8% between T0 and T8 (*p* < 0.001), and by 63.9% between T0 and T14 (*p* < 0.001). The overall decrease in the SCORAD index was greater in Group B even if not significantly. As shown in Table 2, there were no intergroup differences at the various time points evaluated, although apparently there was a trend towards a possibly better clinical improvement in Group B children.

**Table 2:** SCORAD index at the various times of stage 1 in groups A and B, and in stage 2 for groups A (placebo), B (treatment), placebo/placebo (AA) and treatment/treatment (BB) groups

	N	Mean	SD±	% of change *vs* T0	*p* value	
FIRST STAGE						
Group A placebo						
T0	40	13.93	8.28			
T3	37	11.00	11.08	21.0	0.2	
T6	34	10.15	11.71	27.1	0.12	
T8	33	8.15	4.15	41.5	0.001	
T14FU	32	6.53	3.21	53.2	0.0001	
total	177	10.18	8.83			
Group B treatment						
T0	40	14.71	7.65			
T3	35	9.74	10.25	33.8	0.0218	
T6	35	8.00	10.45	45.6	0.0026	
T8	33	6.79	4.08	53.8	0.001	
T14FU	33	5.30	3.82	63.9	0.0001	
total	177	9.17	8.47			
SECOND STAGE						
Group AA placebo/ placebo						
T0		22	6.23	3.13		
T8		22	5.86	6.32	5.9	0.81
T9FU		22	4.64	8.30	25.5	0.41
Group BB treatment/treatment						
T0		27	5.22	3.75		
T8		27	5.07	3.05	2.9	0.87
T9FU		27	3.52	2.59	32.6	0.058

As we had expected, the comparison between the SCORAD groups was not significant (Student’s t test: *p* = 0.29 at T8, and *p* = 0.16 at T14), and therefore this endpoint wasn’t reached.

At the end of the first stage, responders (*n* = 37; AA/BB, i.e., placebo in both stages/treatment in both stages) continued the same treatment as in stage 1 (Table 2). Children classified as non-responders (*n* = 28) were randomly reallocated to placebo or to treatment (AB/BA/AA/BB).

In stage 2, there were no significant decreases in the mean SCORAD index within Group AA at T0, T8 or T9. In Group BB there was a decrease in the SCORAD index of 33% between T0 and T9 (*p* = 0.058) and of 31% between T8 and T9 (*p* = 0.049) (Table 2). In the stage 2 reallocation, we did not analyze the SCORAD index in groups AB and BA because of the low numbers of children in these groups.

### Disease-free interval

A total of 1482 clinical diaries (924 concerning stage 1 and 558 stage 2) were collected. These contained information about subjective symptoms (pruritus and sleep disturbances) and about the use of drugs during the 23 months of the study. As shown in Table 3, pruritus and sleep disturbances decreased, albeit not significantly (*p* = 0.07), in Group A between T0 and T8, while both symptoms were higher, but not significant, between T9 and T14 versus baseline. In Group B, the pruritus score decreased between T0 and T8, and remained stable between T9 and T14. In the same group, sleep disturbances decreased 50% between T0 and T8 (*p* < 0.001), and were absent at T14. However the only statistically significant difference between groups A and B was observed in sleep disturbances at time T14 (*p* = 0.081) (Table 3).

**Table 3:** Analysis of the clinical diaries for itching and sleep disturbances

	T0	T8	T14 FU
Group A placebo			
*Pruritus*			
N	23	23	18
Mean	24.91	19.74	32.61
SD±	22.52	24.11	39.99
*Sleep disturbance*			
N	23	23	18
Mean	9.13	5.87	13.33
SD ±	18.36	16.74	30.24
Group B treatment			
*Pruritus*			
N	21	21	13
Mean	24.33	15.76	15.67
SD±	22.45	17.61	20.33
*Sleep disturbance*			
N	21	21	13
Mean	5.62	2.95	0.00
SD±	14.56	11.27	0.00
*p* value B *vs* A *Pruritus*	0.93	0.53	0.13
*p* value B *vs* A *Sleep disturbance*	0.48	0.50	0.08

We analyzed the use of symptomatic drugs as per time/person expressed in months, which is the sum of the time during which each single child took symptomatic drugs (Table 4). During stage 1, Group A children assumed drugs for 30 months/person between T0 and T8 and between T9 and T14, whereas Group B assumed symptomatic drugs for 16 months/person between T0 and T8 and 8 months/person between T9 and T14. Therefore, Group B assumed drugs between T0 and T8 for less time than did Group A. Moreover, Group B assumed drugs for significantly less time during the T9-T14 period compared with Group A (Fisher’s exact test = 0.001).

**Table 4:** Differences between groups A and B at stage 1 at T0-T8 and T9-T14

Variable	Group A T0-T8	Group B T0-T8	Difference between B-A groups	*P* value	Group A T9-T14 FU	Group B T9-T14 FU	Difference between B-A groups	*p *value
% decrease of the SCORAD index	41.5	53.8	12.3	0.014	19.9	21.9	2.0	0.77
Conventional therapy (months/person)	30	16	−14	0.074	30	9	−21	0.077
% of patients treated with steroids^a^	32.1	23.1	−9.0	0.073	17.9	15.3	−2.6	0.15
% of patients treated with antihistamines^a^	35.7	19.2	−16.5	0.003	32.1	11.5	−20.6	0.028

^a^We considered treated with steroids or antihistamines subjects that took at least one medication during the study. (FU: follow-up)

### Safety and tolerability

Two adverse events were recorded: one in Group A and one in Group B. The one in Group B occurred during stage 1 of the study, between T8 and T14, and consisted in continuous pain in the right leg and difficulty in walking, which did not regress after discontinuation of the treatment. This child was considered a drop out. The analysis of this event using the method reported by Naranjo et al. [21] did not reveal a correlation between the drug and the event. The event recorded in Group A occurred during stage 2 at T3, and consisted of hidradenitis festering. This subject was considered a drop out. The total percent of drop-outs was 25%, and about 70% of subjects left the study during stage 1. The main cause of drop out was difficulty in adhering to the protocol over such a long period.

### Cytokines

In the attempt to shed light on changes that may occur in the immune system during treatment with LDM or placebo, we measured the inflammatory cytokines IL-4, IL-10, IL-12, IL-13 and IFN-γ in children who were willing to undergo sampling during each observation, 45 in stage 1 (22 in Group A and 23 in Group B) and 8 in stage 2 (4 in Group A and 4 in Group B). We did not find changes in the levels of inflammatory cytokines, although there was a wide spectrum of values at the diverse times of observation in both groups. Furthermore, there were no intra- or intergroup differences in cytokine concentration between Group A and Group B, except for a reduction in IL-13 in function of time in both groups (data not shown). Similarly, total IgE levels, the prick test for inhalants, and prick-by-prick for food did not differ between the groups at any time in the two stages.

## Discussion

Little is known about the efficacy of low-dose compounds in eczema [22–27]. In the first stage of this study, disease severity decreased in both groups, although more so in treated than in untreated children (63.9% vs 53.2%) (Table 2). The improvement in the untreated children may be partially explained by the clinical practice of treating the skin of patients using detergents and particular emollients and moisturizers. As shown in Table 2, the improvement of the SCORAD index was even more evident in the group that took the drug in both stages (T0* vs *T9, second stage *p* = 0.058; T8 *vs* T9, second stage *p* = 0.049). These results are in line with the hypothesis that LDM acts by progressively modulating the immune system [16–19] until it reaches homeostasis, which is maintained during follow-up (T9-T14/T8-T9). In fact, Group B maintained a low SCORAD index using much less conventional therapy than Group A during follow-up (Table 4).

From an ethical viewpoint, the placebo group should not have been left without symptomatic drugs; therefore both groups have had access to symptomatic therapy. In both groups the SCORAD reduction was similar, but the consumption of symptomatic drugs was significantly lower in Group B.

The results of the disease-free interval support the hypothesis that LDM acts by progressively modulating the immune system. In fact, the follow-up data showed an improvement in the SCORAD index in the two groups at both stages of the study, but the effect was more pronounced in Group B. Moreover, the finding that itching and sleep disturbances improved in Group A children between T0 and T8, and worsened greatly between T8 and T14 follow-up confirms that eczema is a complex disease in which the psychological aspect plays an important role [7]. Itching and sleep disturbances improved in Group B children between T0 and T8, and between T8 and T14 follow-up thereby confirming the long-term immunomodulation of LDM (Table 3). Subjective symptoms improved in Group B between T0 and T8 and this improvement was maintained during T8-T14 (Table 3). This finding is even more relevant considering that the consumption of symptomatic drugs was 30% lower in Group B than in Group A (9 months/person *vs* 30 months/person; *p* = 0.001) (Table 4) with an improvement in the family management of eczema according to the parents and children during control visits. Sleep disturbances improved greatly (T0/T8 first stage *p* < 0.001) in Group B, and were absent in the second stage (Table 3). This is in line with the well known effect of IFN-γ in promoting REM or NREM sleep [28–30].

Interferons and cytokines at the concentration of 10 fg/ml, which is a physiological concentration, represent “signaling molecules” able to exert immunomodulatory activity on target cells [16–19]. In a condition of imbalance of the immune system, these biological molecules might be able to restore the balance in over-expressed lymphocyte clones [14, 16].

A critical point of “signaling molecules” (and peptides in general) oral administration is represented by their low bioavailability, which is typically less than 1–2%: for this reason an effective drug delivery system (SKA) is required in order to improve this key parameter. Specific studies about the stability of cytokines at intestinal pH have never been conducted, nevertheless the abundance of data produced about the pharmacological activity of those oral administered molecules in sub-nanomolar concentrations enhances the hypothesis of a mechanism overcoming the gastro-intestinal barrier. Moreover, it is difficult to investigate the pharmacokinetics of low-dose orally administered cytokines. The limits of sensitivity of current assay techniques do not allow to perform pharmacokinetic tests following the oral administration of the above mentioned proteins at the usual concentrations (fg/ml). However Tessaro et al. reported that an oral administration of a low dose of rhFSH is able to ameliorate some peculiar features of PCOS (Polycystic Ovary Syndrome) in hyper-androgenized mice [31]. Martin-Martin LS et al. has shown in an open randomized active controlled clinical trial the activity of low-dose SKA IL-4, IL-10, and Ab anti IL-1 versus DMARDs in maintaining low disease activity in patients affected by rheumatoid arthritis in remission after therapy with biological drugs [32].

SKA low dose molecules work by bringing to the system information able to activate auto-regulation mechanisms. This immunomodulating effect could underlie the decrease in the SCORAD index and the clinical improvements seen in our study. In particular, IFN-γ is a powerful modulator of the Th2 lymphocyte clone, and IL-12 is a strong signal of induction of IFN-γ. In addition, IL-4 and IFN-γ mirror the Th1/Th2 imbalance and the state of inflammation of eczema. Consequently, the expansion of the Th2 lymphocyte clone can be regulated by administration of low-dose SKA IFN-γ and IL-12 [14–19]. Consequently, we studied the immunomodulatory effect of IFN-γ and IL-12 low-dose SKA. We did not identify a linear variation between blood levels of inflammatory cytokines and the clinical effects observed after administration of low-dose SKA IFN-γ and IL-12 (data not shown). Similarly, we were unable to identify changes in the blood concentrations of these molecules that could support the clinical results, probably because of their brief half life and the very low concentration at which they were administered.

In terms of pharmacoeconomics, the advantages of LDM treatments for chronic eczema are compelling, even though it is difficult to compare the costs of a topical/oral symptomatic therapy to an immunomodulating one.

Since LDM therapies are mainly aimed at recovering the homeostasis of the entire system, the need of symptomatic therapies for the management of different and numerous symptoms results to be much lower, as emerged by this clinical trial (Table 3). We can furthermore affirm that the Group B uses the symptomatical treatment in a significantly reduced measure compared to the Group A, even in the follow-up period (Disease Free Interval) (Table 4), this data suggests that low dose treatment can be given according to a reduced therapeutic cycle model.

A limitation of our study is the relatively large number of drop-outs. This was probably due to the long-term nature of the study (nearly 2 years), which, although potentially enabling us to obtain robust clinical data, may have been a deterrent to compliance.

## Conclusion

Preliminary evidence suggests potential benefit, but further work is needed to validate this approach. However, in this study, itching, sleep disturbances, and eczema exacerbations were reduced in children treated with LDM compared with conventional therapy, and LDM was well tolerated. Low-dose medicine had good long-term clinical efficacy (reduction of skin lesions, itching and sleeplessness); its benefit persisted during follow-up; it resulted in a reduced intake of symptomatic therapy, was well tolerated and had a good safety profile.

Although there is a need for studies with a more streamlined design to increase compliance and reduce the number of drop-outs, and to obtain more information about the clinical and immunological activity of LDM, the latter can be considered a valid support to conventional treatment in some cases of chronic eczema of mild to moderate intensity.

## Abbreviations

ELISA: Enzyme-linked immunosorbent assay
IFN-γ: Interferon-γ
IgE: Immunoglobulin E
IL-12: Interleukin-12
LDM: Low-dose medicine
NREM: Non- rapid eye movement
REM: Rapid eye movement
SCORAD: Scoring atopic dermatitis
SKA: Sequential kinetic activation
